# Improved outcome of ^131^I-mIBG treatment through combination with external beam radiotherapy in the SK-N-SH mouse model of neuroblastoma

**DOI:** 10.1016/j.radonc.2017.05.002

**Published:** 2017-09

**Authors:** Aurélien Corroyer-Dulmont, Nadia Falzone, Veerle Kersemans, James Thompson, Danny P. Allen, Sarah Able, Christiana Kartsonaki, Javian Malcolm, Paul Kinchesh, Mark A. Hill, Boris Vojnovic, Sean C. Smart, Mark N. Gaze, Katherine A. Vallis

**Affiliations:** a CRUK/MRC Oxford Institute for Radiation Oncology, Department of Oncology, Oxford University, UK; b Nuffield Department of Population Health, Oxford University, UK; c University College London Hospitals NHS Foundation Trust, London, UK

**Keywords:** Radiotherapy, ^131^I-mIBG, Neuroblastoma, Image-guided radiotherapy

## Abstract

**Purpose:**

To assess the efficacy of different schedules for combining external beam radiotherapy (EBRT) with molecular radiotherapy (MRT) using ^131^I-mIBG in the management of neuroblastoma.

**Materials and methods:**

BALB/c nu/nu mice bearing SK-N-SH neuroblastoma xenografts were assigned to five treatment groups: ^131^I-mIBG 24 h after EBRT, EBRT 6 days after ^131^I-mIBG, EBRT alone, ^131^I-mIBG alone and control (untreated). A total of 56 mice were assigned to 3 studies. Study 1: Vessel permeability was evaluated using dynamic contrast-enhanced (DCE)-MRI (*n* = 3). Study 2: Tumour uptake of ^131^I-mIBG in excised lesions was evaluated by γ-counting and autoradiography (*n* = 28). Study 3: Tumour volume was assessed by longitudinal MR imaging and survival was analysed (*n* = 25). Tumour dosimetry was performed using Monte Carlo simulations of absorbed fractions with the radiation transport code PENELOPE.

**Results:**

Given alone, both ^131^I-mIBG and EBRT resulted in a seven-day delay in tumour regrowth. Following EBRT, vessel permeability was evaluated by DCE-MRI and showed an increase at 24 h post irradiation that correlated with an increase in ^131^I-mIBG tumour uptake, absorbed dose and overall survival in the case of combined treatment. Similarly, EBRT administered seven days after MRT to coincide with tumour regrowth, significantly decreased the tumour volume and increased overall survival.

**Conclusions:**

This study demonstrates that combining EBRT and MRT has an enhanced therapeutic effect and emphasizes the importance of treatment scheduling according to pathophysiological criteria such as tumour vessel permeability and tumour growth kinetics.

Neuroblastoma is the most frequently occurring extra-cranial tumour in early childhood [1]. It is risk stratified at diagnosis by age, stage and molecular pathology into low-, intermediate- and high-risk groups. Despite major advances in the development of anti-cancer agents and the use of multi-modal therapeutics in the treatment of this disease [1], only modest progress has been made in the last decade to improve the survival of children with high risk neuroblastoma (HRNB) [2]. External beam radiotherapy (EBRT) to the primary tumour site is part of the standard treatment protocol for HRNB, and has been shown to improve local control and survival [1]. Molecular radiotherapy (MRT) with Iodine-131 meta-iodobenzylguanidine (^131^I-mIBG), a noradrenaline analogue taken up by neuroblastomas, phaeochromocytoma and paragangliomas that overexpress the noradrenaline transporter, has long been used for the treatment of refractory or relapsed neuroblastoma, and has also been incorporated into induction and consolidation regimens [3]. However, the optimal use and timing of ^131^I-mIBG in the management of neuroblastoma remains unclear [3], and there is no consensus regarding the concomitant use of chemotherapy, radiation sensitizers or EBRT.

Combined modality EBRT plus MRT for HRNB treatment could prove to be an effective addition to currently available therapeutic options. The distinct advantage of combining different radiation therapy modalities lies in the ability to achieve higher radiation absorbed tumour doses without compromising the dose limiting organs of each individual therapy [4], [5]. The efficacy of combining EBRT and ^131^I-mIBG in the management of malignant phaeochromocytoma and paraganglioma was recently demonstrated by Fishbein and co-workers [6]. However, an effective combination was not defined despite the different treatment schedules evaluated. Several studies have investigated the rationale of combining EBRT and MRT *in vitro* for glioblastoma and head and neck squamous cell carcinoma [7], [8] as well as *in vivo* for breast, colorectal and squamous cell carcinoma models [9], [10], [11], showing an additive effect for the combination of the two treatments when they are given sequentially or concurrently. Additionally, it has been shown that EBRT increases vessel permeability which could increase MRT uptake and distribution within the tumour [4]. The challenge is to define more effective approaches for the combination of EBRT and MRT in the treatment of HRNB while maintaining an acceptable toxicity profile.

The aim of this study was to explore the interaction between EBRT and MRT *in vivo* and to provide a framework for combination treatment protocols of neuroblastoma. To achieve this, three studies were undertaken. In study 1 dynamic contrast-enhanced (DCE)-MRI was used to assess the effect of EBRT on vessel permeability and its impact on ^131^I-mIBG uptake and distribution within the tumour. To address this phenomenon, in study 2, ^131^I-mIBG tumour uptake and radiation absorbed dose were analysed with or without prior EBRT treatment. In addition, the scheduling of EBRT before and after MRT was considered. For the latter, the temporal separation between EBRT and MRT was selected to coincide with the time-point at which tumour regrowth was observed after initial MRT. Finally, in study 3 the efficacy of the specific combined schedules of EBRT and MRT was evaluated by assessing tumour volume and overall survival with longitudinal MR imaging.

## Materials and methods

### Cell line

The SK-N-SH human neuroblastoma cell line (American Type Culture Collections, Manassas, VA, USA) was cultured in Dulbecco’s Modified Eagle’s Medium (DMEM, Sigma–Aldrich, UK) supplemented with 10% foetal calf serum (FCS) (Invitrogen, UK), 2 mM glutamine (Sigma–Aldrich, UK) and 100 U/mL penicillin/streptomycin (Invitrogen, UK).

### Mouse neuroblastoma model

**Fig. 1:**
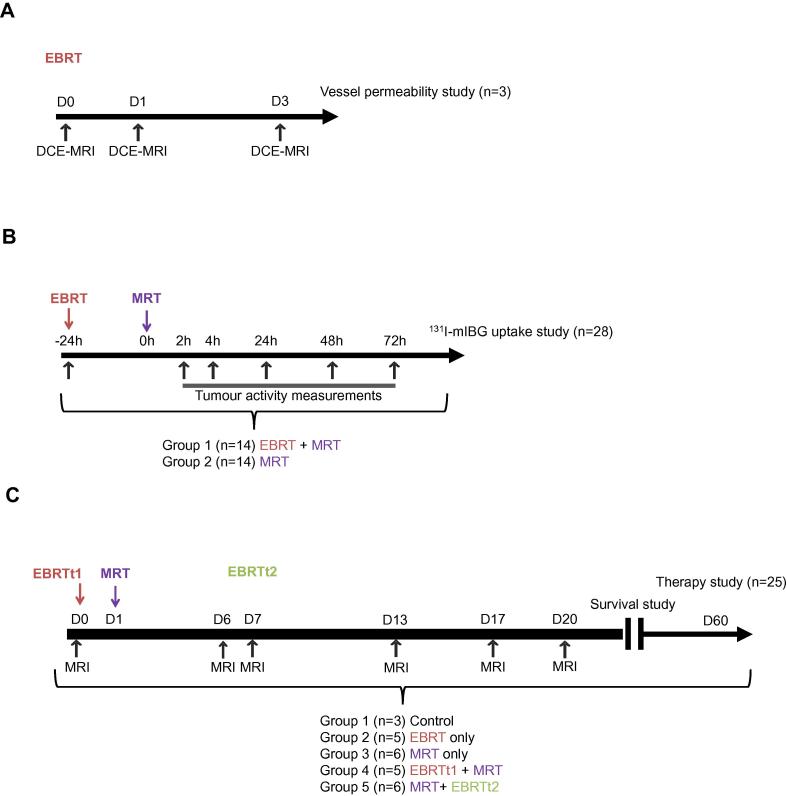
Experimental schema for (A) Study 1, vessel permeability, (B) Study 2, ^131^I-mIBG tumour uptake and (C) Study 3, tumour growth inhibition.

All animal procedures were carried out in accordance with the UK Animals (Scientific Procedures) Act 1986 and with local Ethics Committee approval. In preliminary experiments the success rate of tumour development following subcutaneous injection of SK-N-SH cells into the flanks of BALB/c nu/nu mice was found to be low (10%). Therefore, SK-N-SH cells (5 × 10^6^) in 100 µL of matrigel were injected subcutaneously into the flanks of Nod *scid* gamma mice (20–25 g, eight weeks old, female; Charles River, UK). In this mouse strain the success rate of xenograft development was 100%. Tumours were excised and homogenized when they reached a volume of 1 cm^3^ (at approximately five weeks). Tumour homogenates were injected into the right flank of recipient BALB/c nu/nu mice (*n* = 56) (15–20 g, eight weeks old, female; Charles River, UK). Following this procedure the proportion of BALB/c nu/nu mice that developed xenograft tumours was 100%. Xenografts grew to an average volume of 500 mm^3^ (range 428–532 mm^3^) by 7 weeks following subcutaneous inoculation of SK-N-SH homogenate. The 56 recipient BALB/c nu/nu mice were then assigned to three sub-study groups as shown in Fig. 1A–C (*n* = 3; *n* = 28 and *n* = 25).

### Magnetic resonance imaging (MRI)

MRI was performed using a 4.7 T 310 mm horizontal bore Varian Nuclear Magnetic Resonance Spectrometer (VNMRS; Varian Inc., Palo Alto, CA, USA) preclinical imaging system as previously described [12]. For all imaging, the mouse was placed in the supine position. Respiration was monitored using a pressure-sensitive balloon around the abdomen. A T2w sequence (fast spin echo multislice, TE = 22.43 ms, TR = 1556.49 ms, slice thickness = 0.33 mm, 58 slices, respiratory gated) was used for tumour detection and is subsequently referred to as “anatomical MRI”. Transport of animals between MRI and EBRT treatment rooms took less than a minute and animals remained anaesthetised during transport. For all experiments, mice were maintained under anaesthesia: 4% isoflurane for induction, 2% for maintenance in air supplemented with oxygen (70%/30% v/v). Imaging was performed consecutively.

### External beam radiation therapy

EBRT treatments (220 kVp X-rays; half-value layer of 0.93 mmCu; 2 Gy/min; 14 mm × 7 mm field at the isocentre) were given using a small animal radiation research platform (SARRP) irradiator, (Xstrahl Ltd, Camberley, Surrey, UK). Dosimetric measurements for this collimator were performed using EBT3 film (Ashland ISP Advanced Materials, Wayne, NJ) that was calibrated against absolute measurements following the recommendations of the report of the American Association of Physicists in Medicine Task Group 61 [13]. MR images were used to identify and delineate the tumour volume using a custom made cradle [12], which allowed animals to be transported and mounted on the SARRP irradiator with minimal movement between procedures. A cone beam CT (CBCT) image was acquired using the SARRP and co-registered with the MR image using in-house MATLAB® (MathWorks, Natis, MA, USA) software based on the Modality Independent Neighbourhood Descriptor (MIND) algorithm for multi-modal deformable registration to compensate for the non-linear spatial distortions inherent in MR imaging [14] (see Supplementary Fig. 1). Treatment planning and subsequent beam delivery was performed using Muriplan (Xstral Ltd, Camberley, Surrey, UK) [15], with segmentation performed using the CBCT image; and targeting and planning using the combined MRI-CBCT image.

### Study 1: Vessel permeability

To evaluate blood vessel permeability following EBRT, three tumour-bearing mice (*n* = 3) underwent DCE-MRI (Fig. 1A). The effect of EBRT on tumour and vessel permeability was evaluated before EBRT, and 24 h and 72 h after EBRT by DCE-MRI. Muscle on the opposite side to the tumour, outside of the irradiated volume, was used for comparison. Respiration-gated 3D gradient echo imaging (TE = 0.55 ms, TR = 1.1 ms, flip angle 5 degrees) covering a field of view of 54 × 27 × 27 mm at an isotropic resolution of 0.42 mm was used. Gadodiamide (Omniscan®, GE Healthcare) (30 µL, 0.5 M) was infused via a tail vein cannula, and uptake was monitored every 9 s up to 431 s post injection (p.i.) using 60 repetitions.

### Study 2: ^131^I-mIBG tumour uptake and dosimetry

^131^I-mIBG (20 MBq) was administered to two groups of xenograft-bearing mice (*n* = 28), the time schedule for tumour activity measurement is shown in Fig. 1B. In one group (EBRT + MRT), mice received EBRT 24 h prior to ^131^I-mIBG and in the other group MRT was given alone. At set time points following injection, tumours were excised (*n* = 3 at 2, 4, 24 and 48 h p.i. and *n* = 2 at 72 h p.i.) and the accumulated radioactivity in tumours was measured in both groups using a gamma counter (2480 WIZARD, Perkin Elmer, Waltham, MA, USA). Tumour cryo-sections from the two groups 24 h after ^131^I-mIBG injection were also prepared for autoradiography. Sections were left overnight to expose an image phosphor plate and analysed using a Cyclone® Plus Storage Phosphor System (Perkin Elmer, Shelton, CT, USA).

The Medical Internal Radionuclide Dose (MIRD) schema was used to calculate the absorbed radiation dose to the tumour. Time activity curves for the 2 groups were generated from gamma counter radioactivity measurements of each tumour at the selected time points. Cumulated activity was assessed by numerical integration of the time activity curve per time point by using a trapezoidal integration method and assuming physical decay after the last data point. The dose to the target area per unit cumulated activity in the source region, i.e. *S*-values, were determined from event-by-event Monte Carlo (MC) transport with the general-purpose code PENELOPE [16] based on the unabridged nuclear decay data of ^131^I [17]. Simulations were run in water (mass density *ρ* = 1 g cm^−3^) for a sphere of equivalent tumour volume. For validation, PENELOPE simulation results were compared to OLINDA/EXM single sphere values [18] (Supplementary Table 1).

### Study 3: Therapy

For the main tumour growth inhibition study (Fig. 1C), animals were randomly assigned to one of the following 5 treatment groups (*n* = 25): Control (*n* = 3), EBRT (*n* = 5), MRT (*n* = 6), EBRTt1 + MRT (*n* = 5) and MRT + EBRTt2 (*n* = 6). The group designated EBRTt1 + MRT received MRT 24 h following EBRT and the group designated MRT + EBRTt2 received MRT 6 days before EBRT. The scheduling of the MRT + EBRTt2 group was based on a prior study showing a regrowth of tumour 6 days after MRT [19]. All mice underwent MRI imaging to measure tumour volume at baseline and at days 6, 7, 13, 17, 20 and then at weekly intervals thereafter until a pre-defined humane endpoint, a maximum tumour volume of 800 mm^3^, was reached. Mice were then euthanised. For animals with complete tumour regression, 53 days after the end of treatment was defined as an arbitrary censoring time for overall survival.

#### EBRT

A single fraction of 5 Gy using the SARRP irradiator, based on previously published experience of the SK-N-SH model [19], was administered.

#### MRT

For all MRT treatments, ^131^I-mIBG (GE Healthcare, UK) was administered at a specific activity of 185 MBq/mL. Animals received intravenous (i.v.) tail vein injections of vehicle (1% benzyl alcohol, 100 µL) or ^131^I-mIBG (20 MBq, 100 µL) [20]. Mice were fed *ad libitum* and potassium iodine (0.02%, KI, Sigma–Aldrich) was added to the drinking water on the day of treatment and for 7 days thereafter to block thyroid uptake of the radiolabelled iodine.

### Image processing and analysis

ImageJ (version 1.50f) software was used for all image processing and analysis detailed below [21]. Tumour volume delineation was performed manually on all adjacent T2w slices. For DCE-MRI, T1 signal within the muscle and the tumour was analysed prior to and during Gadodiamide injection. The tumour volume was calculated by multiplying the area of the tumour on each slice by the slice thickness and then adding these together.

### Statistical analyses

Change in vessel permeability, is presented as mean ± SD (Standard Deviation), and statistical significance is reported at the 5% level using a standard t-test. Analysis of variance F-tests were used to assess tumour volume differences between treatment groups. Kaplan–Meier curves were used to assess survival and a Weibull model was fitted to assess the effects of treatment on survival with quasi-variances. Statistical analyses were done using GraphPad Prism® (version 5, GraphPad Software, Inc, USA) and R software (version 3.3.1, R Foundation for Statistical Computing, Vienna, Austria) [22].

## Results

### Studies 1 and 2: Effect of EBRT on vessel permeability and ^131^I-mIBG tumour uptake

**Fig. 2:**
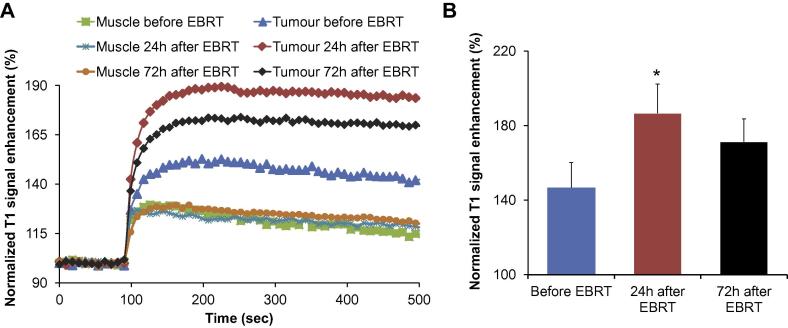
MRI assessment of EBRT treatment effect on the tumour vascular permeability and ^131^I-mIBG uptake. (A): Normalised T1 signal enhancement curves during Gadodiamide injection in healthy tissue and tumour at different time points: before (blue), 24 h (red) and 72 h (black) after EBRT treatment. (B): Quantitative normalised T1 signal enhancement. Mean ± SD, *n* = 3. *p < 0.05 *versus* before EBRT.

DCE-MRI was performed at baseline, 24 and 72 h following EBRT to assess tumour vessel permeability and mean normalized T1 signal enhancement is reported for *n* = 3 animals (Fig. 2). As shown in Fig. 2A, there was an initial increase in the normalised T1 signal as a measure of vessel permeability in tumour tissue compared to unirradiated normal tissue (muscle). The normalized T1 signal expresses the T1 enhancement as a function of the baseline condition (signal before injection). Vessel permeability increased significantly 24 h after EBRT compared to the baseline (*p* < 0.05). By 72 h the mean signal enhancement has diminished compared to 24 h, although the difference between the 24 h and 72 h time points does not reach statistical significance (Fig. 2B). DCE-MRI results for individual mice are shown in Supplementary Fig. 1 to clearly demonstrate this trend. Results from this study showed that the optimal interval between EBRT and administration of MRT was 24 h. This information was used to evaluate the effect on MIBG uptake following EBRT exposure 24 h prior to MRT *versus* MRT alone.

**Fig. 3:**
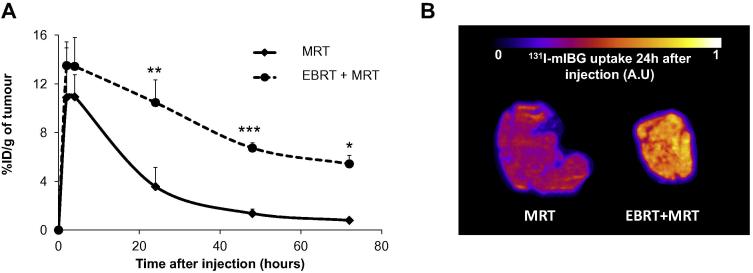
EBRT treatment effect on ^131^I-mIBG uptake. (A) ^131^I-mIBG uptake with or without EBRT pretreatment at 2 h, 4 h, 24 h, 48 h and 72 h after ^131^I-mIBG injection. Mean ± SD, *n* = 3 for each group and each time point except for the 72 h time point (*n* = 2). *p < 0.05, **p < 0.01 and ***p < 0.001 *versus* MRT group. (B) Autoradiography showing ^131^I-mIBG uptake in tumour following MRT alone or 24 h after EBRT treatment (arbitrary units, A.U.).

The percentage of injected ^131^I-mIBG dose per gram of tumour tissue (%ID/g) was slightly but not statistically significantly increased 2 and 4 h p.i. in the EBRT + MRT group (where MRT administration was preceded by EBRT 24 h earlier) in comparison to the MRT only group (%ID/g at 2 and 4 h: 13.50 ± 1.94 and 13.43 ± 2.37 *versus* 10.89 ± 4.06 and 10.94 ± 1.80 respectively) (Fig. 3A). However, ^131^I-mIBG uptake was significantly greater in the EBRT + MRT group at 24 h (*p* < 0.01), 48 h (*p* < 0.001) and 72 h (*p* < 0.05) compared to the MRT only group (%ID/g at 24, 48 and 72 h: 10.47 ± 1.86, 6.73 ± 0.43 and 5.43 ± 0.69 for EBRT + MRT group *versus* 3.57 ± 1.57, 1.36 ± 0.36 and 0.80 ± 0.15 for the MRT only group). Autoradiography was performed on two tumours that were excised 24 h after administration of ^131^I-mIBG, one each from the EBRT + MRT and MRT groups. Autoradiography showed a marked increase in tumour-associated ^131^I when EBRT was administered prior to MRT compared to MRT alone (Fig. 3B).

PENELOPE simulated absorbed fractions for unit density spheres were within 10% of OLINDA/EXM self-irradiation values (Supplementary Table 1). Differences in sphere *S*-values can be ascribed to the different MC codes used [23]. Dose calculations based on time activity curves and PENELOPE MC simulation results showed an increase in dose attributable to MRT in tumours when they were also treated by EBRT 24 h before MRT compared to MRT alone (8.03 and 3.61 Gy respectively – Supplementary Table 2). Taken together the results of the DCE-MRI and tumour uptake studies informed the decision to administer ^131^I-mIBG 24 h after EBRT treatment in the EBRTt1 + MRT group.

### Study 3: Therapy study

**Fig. 4:**
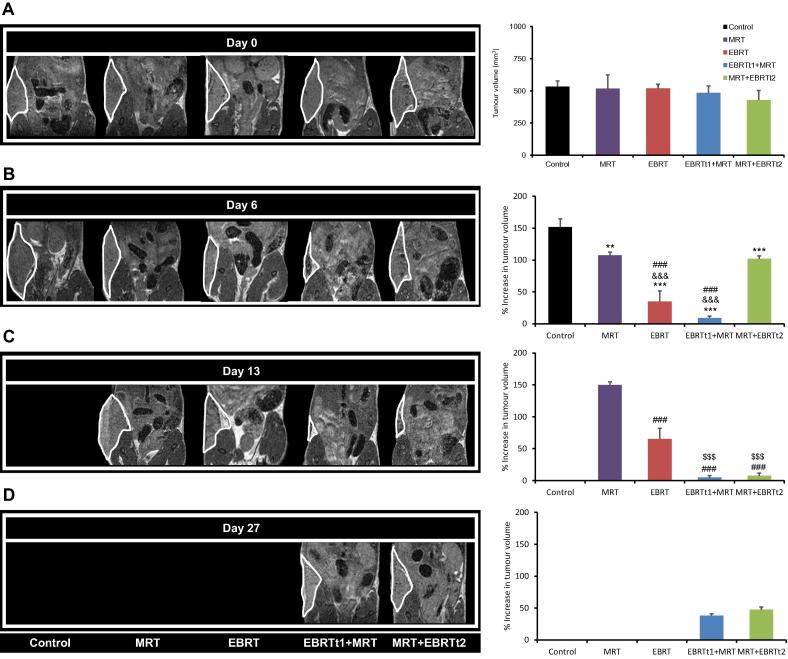
MRI evaluation of the tumour volume. Left panels: Representative T2w MRI images for the 5 different groups of mice before treatment, D0 (A), and at late times, D6 (B), D13 (C), D27 (D), after the administration of the treatments. Right panels: Quantitative analyses of tumour volume at D0 and the percentage of increase or decrease of tumour volume in comparison to pretreatment at D6, D13 and D27. Mean ± SD, *n* = 3 for the Control group, *n* = 5 for the EBRT alone and EBRTt1 + MRT groups and *n* = 6 for MRT alone and MRT + EBRTt2 groups. **p < 0.01 and ***p < 0.001 *versus* Control group, ^###^*p* < 0.001 *versus* MRT group, ^&&&^*p* < 0.001 *versus* MRT + EBRTt2 and ^$$$^*p* < 0.001 *versus* EBRT alone group for time effect.

**Fig. 5:**
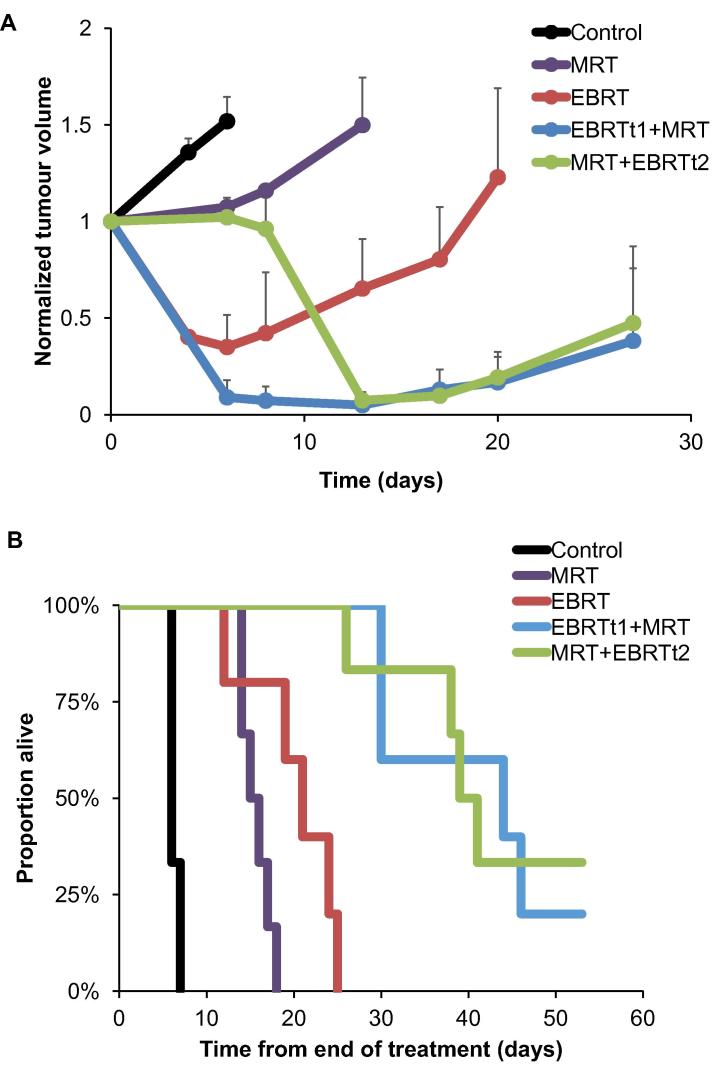
Effect of radiotherapy treatments and scheduling on (A) SK-N-SH tumour volume and (B) overall survival. (A) Normalised tumour volume evolution. Mean ± SD, *n* = 3 for the Control group, *n* = 5 for the EBRT alone and EBRTt1 + MRT groups and *n* = 6 for MRT alone and MRT + EBRTt2 groups. (B) Kaplan-Meier curves of the proportion of animals reaching the endpoint for tumour volume evaluation.

There was no significant difference in tumour volume for the 5 treatment groups at baseline (Day 0) (Fig. 4A). Tumour progression was rapid in the control group with all tumours reaching the maximum allowed volume of 800 mm^3^ in 7 days. As shown in Fig. 4B, ^131^I-mIBG alone caused a significant delay in tumour progression in comparison to the control group (*p* < 0.01) as tumour volume was stable during the first 5 days p.i. Regrowth of one tumour was observed at Day 7 and this tumour reached 800 mm^3^ by Day 13 (Fig. 4B and Fig. 5A). In contrast, EBRT alone caused a rapid and significant regression in tumour volume (65.00 % ± 16.67 decrease) compared to control (51.84% ± 12.66 increase), MRT (7.45% ± 4.82 increase) and MRT + EBRTt2 (2.15% ± 4.35 decrease) groups at Day 6 (*p* < 0.001) (Fig. 5). Additionally, one recurrence was observed after Day 6 and although a significant difference (*p* < 0.001) in tumour volume compared with the MRT group at Day 13 was observed (Fig. 4C), the volume of the recurrent tumour reached 800 mm^3^ at Day 25 after EBRT alone (Fig. 5A). The combined effect of EBRT delivered 1 day prior to MRT (EBRTt1 + MRT group), resulted in a significant reduction of tumour volume (91.04% ± 2.94 decrease) between Day 0 and Day 6; *p* < 0.001 *versus* all the other groups (Fig. 4A–C). Similarly, the combination of EBRT administered after MRT (MRT + EBRTt2) also resulted in a significant decrease in the tumour volume by Day 13 in comparison to the MRT and EBRT only groups (*p* < 0.001). As for the EBRTt1 + MRT group, animals in the MRT + EBRTt2 group developed tumour regrowth from Day 17 (Fig. 5A). To evaluate treatment efficacy, Kaplan–Meier curves were calculated with time from start of treatment to the time at which the permitted upper limit of tumour volume (800 mm^3^) was reached, as the time scale. For the EBRTt1 + MRT and the MRT + EBRTt2 groups, one mouse (1/5), and two mice (2/6), respectively, survived to the end of the observation period. As shown in Fig. 5B, the survival benefit of EBRT and MRT treatment alone was highly significant compared to the untreated group (*p* < 0.001). EBRT had a significant survival benefit compared to MRT (*p* = 0.03). Survival for each combined therapy group was significantly higher compared to the control group (*p* < 0.001) and to the EBRT and MRT alone groups (*p* < 0.001 and *p* < 0.001, respectively) (Fig. 5B). There were no significant differences in survival between the two combined treatment groups.

## Discussion

The potential benefits of combining EBRT and MRT are substantial as the dose limiting organs of the two modalities differ, thus allowing escalation of solid tumour doses without exceeding dose to the limiting organs [4]. This approach is of particular interest in the treatment of neuroendocrine tumours in paediatric patients considering the age of the affected patients and the location of this cancer (frequently adjacent to dose-limiting normal organs such as the kidney and liver) [24]. However, so far, the optimal regimen to combine the two therapies remains elusive.

It has been suggested that when MRT is given as the first treatment, the intratumoural distribution of ^131^I-mIBG could be used to inform the planning of subsequent EBRT [4], [5]. For example, EBRT delivery could be optimized by using intensity-modulated radiotherapy (IMRT) to address the inhomogeneous absorbed dose distribution typical of MRT that results from non-uniform intratumoural distribution of radioactivity. Although this approach has several merits, it presents a clinical challenge in the management of a radioactive patient during EBRT treatment. In addition it may not be possible to proceed with EBRT immediately after administration of MRT since patients often require stem cell rescue following ^131^I-mIBG. However, it is well documented that MRT given concurrently with EBRT is more effective than when given after a prolonged interval [25], [26], [27]. Therefore if it were possible to separate the two radiation modalities without affecting their combined efficacy it would avoid added complexities in the clinical management of paediatric patients. To this end we evaluated two regimens that could potentially be clinically adopted without complicating patient management. Our choice of scheduling, EBRT 24 h before MRT and MRT followed by EBRT after an interval of 7 days was informed by pre-clinical evaluation of ^131^I-mIBG uptake and its effect on tumour volume as assessed by MRI imaging.

Several studies have noted enhanced accumulation of targeted radionuclides as a result of increased vessel permeability following EBRT [5], [28], [29], [30]. In this study, vessel permeability was evaluated by DCE-MRI after EBRT to elucidate the optimal time for ^131^I-mIBG treatment. The results show that for the SK-N-SH human neuroblastoma model, a significant increase in vessel permeability occurred 24 h after EBRT treatment. This finding allowed us to propose a schedule for the combined treatment of EBRT 24 h before MRT treatment (EBRTt1 + MRT) to potentiate the effect of both treatments. The combined treatment led to a more than two fold increase in ^131^I-mIBG uptake and radiation absorbed dose than that attributable to MRT only (8.03 and 3.61 Gy with or without EBRT beforehand). The increased vessel permeability was observed only in tumour tissue, as the normalized T1 signal in muscle remained the same over the selected time course (Fig 2A). These results suggest that with prior highly conformal EBRT, it would be possible to increase the radiation absorbed dose attributable to MRT within the tumour without increasing dose to un-irradiated healthy tissue. Furthermore, tumour shrinkage caused by prior EBRT resulted in an increased uptake and therefore absorbed dose of MRT compared to the MRT only group. Bearing in mind that the optimal cure diameter range for ^131^I is 2.6–5.0 mm [31], a regimen that allows for tumour shrinkage before MRT would increase the efficacy of the treatment.

The rationale for the schedule of the second combined treatment group (MRT first followed by EBRT at Day 7) was based on the differential between dose- and repair- rates of the tumour following ^131^I-mIBG induction [32]. During the 7 day period after ^131^I-mIBG treatment, radiation induced damage appears to be in equilibrium with tumour growth as no progression was observed. However, we speculate that when the dose rate from ^131^I-mIBG fell below the repair rate, tumour regrowth (repopulation) was observed. If EBRT is initiated at this point then there is no “wastage” of the cytocidal effect conferred by ^131^I-mIBG treatment. Despite the increased ^131^I-mIBG uptake after EBRT in the EBRTt1 + MRT group, this did not translate into a significant effect on survival when compared to the MRT + EBRTt2 group. Both treatment regimens (EBRTt1 + MRT and MRT + EBRTt2) significantly increased survival compared with either therapy alone and compared to the control group. Nonetheless, as previously reported, there was a heterogeneous treatment response to the combined therapies with 20% (1/5) and 33% (2/6) of the animals in the EBRTt1 + MRT and MRT + EBRTt2 groups respectively tumour free 53 days after the end of the respective treatments. The small number of animals used in each group could potentially limit the interpretation of results. In addition, there are a large number of biophysical factors that may contribute to the observed variability in the tumour response [33]. Tumour size at the start of therapy could potentially affect the efficacy of MRT treatment. Interestingly, the differences in tumour size between the EBRTt1 + MRT and MRT + EBRTt2 groups at the time of MRT administration did not have a significant effect on overall survival. EBRT treatment before MRT (EBRTt1 + MRT) was effective in reducing the tumour size resulting in a greater uptake of ^131^I-mIBG, and it is also plausible that the sensitising effect of low dose ^131^I-mIBG treatment before EBRT contributed to the efficacy of the MRT + EBRTt2 combination.

In this study, we had not set out to establish dose equivalence between EBRT and MRT, primarily as the focus was the establishment of a combined schedule for EBRT and MRT informed by vessel permeability, MRT uptake and tumour growth. Dosimetry could be improved by quantitative small animal imaging which was not possible at the time of this study. Furthermore, for a curative intent, fractionation of both EBRT and MRT should be considered in the future, taking into consideration tissue toxicity.

In conclusion, combinations of EBRT and ^131^I-mIBG therapy are more effective than either EBRT or MRT alone, with overall survival approximately equal to the sum of the two effects. In this study no statistical difference between the overall survival was observed when the order of treatments in the combined regimens was swapped. We propose that, with a prior knowledge of pathophysiological criteria such as vessel permeability and tumour repopulation kinetics, it would be possible to develop patient-specific EBRT and ^131^I-mIBG regimens that can promote long term local control of bulky tumours with focal EBRT augmented by ^131^I-mIBG. This strategy could potentially be used to develop a prospective protocol for multi-modality management of HRNB in paediatric patients.

## Conflict of interest statement

The authors have no conflict of interest to disclose regarding this study.
