# Supplementary material for: Improved outcome of 131I-mIBG treatment through combination with external beam radiotherapy in the SK-N-SH mouse model of neuroblastoma
Source: Radiother Oncol. 2017 Sep;124(3):488–95. doi: 10.1016/j.radonc.2017.05.002 (PMC5636618; doi:10.1016/j.radonc.2017.05.002)
Supplement: Supplementary data [file mmc1.pptx]

## Slide 1
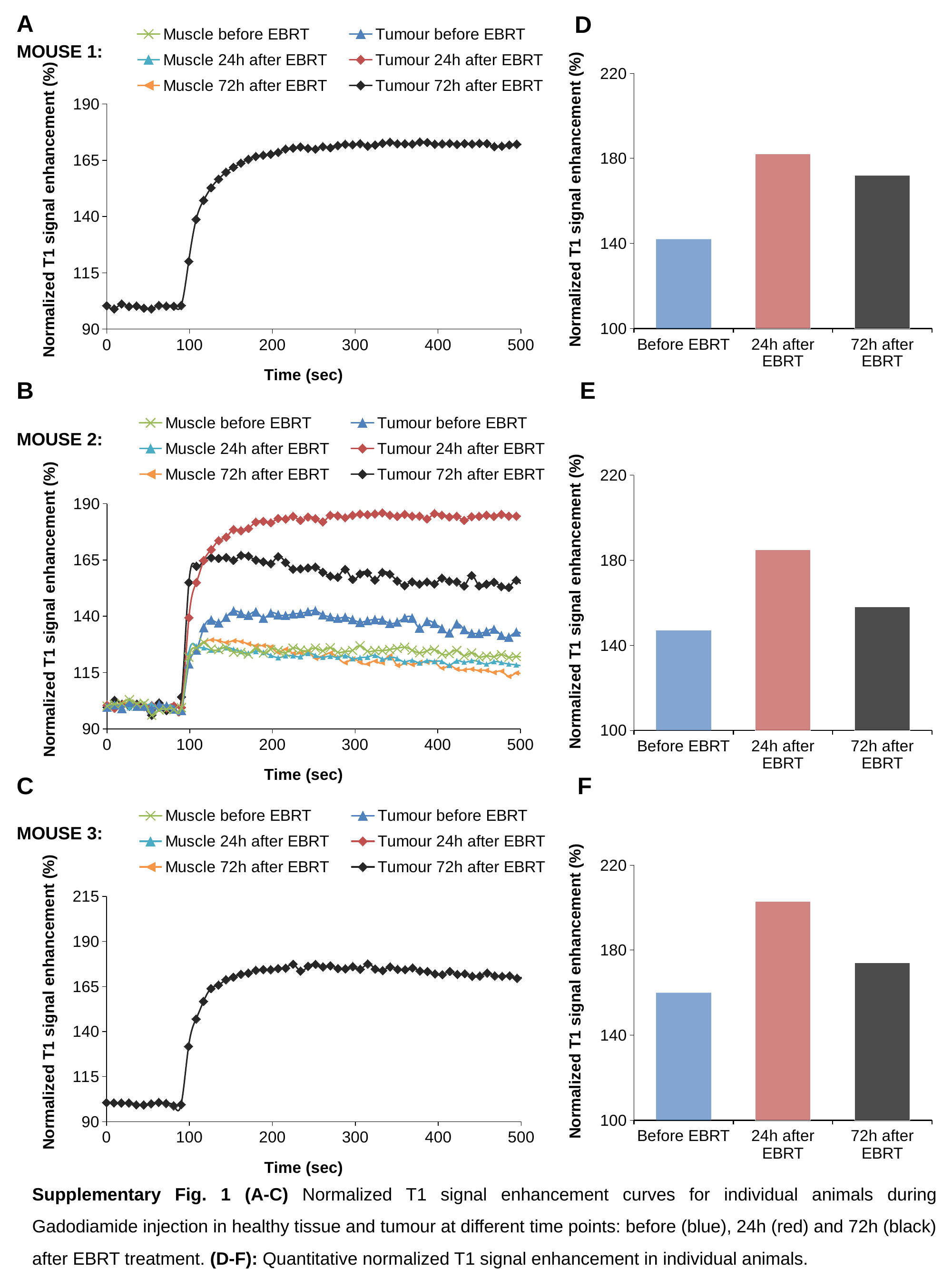

### Chart
| Category | | | | | | |
|---|---|---|---|---|---|---|
### Chart
| Category | |
|---|---|
| Before EBRT | 142.0 |
| 24h after EBRT | 182.0 |
| 72h after EBRT | 172.0 |A
D
MOUSE 1:
B
E
### Chart
| Category | |
|---|---|
| Before EBRT | 147.0 |
| 24h after EBRT | 185.0 |
| 72h after EBRT | 158.0 |
### Chart
| Category | | | | | | |
|---|---|---|---|---|---|---|MOUSE 2:
C
F
### Chart
| Category | |
|---|---|
| Before EBRT | 160.0 |
| 24h after EBRT | 203.0 |
| 72h after EBRT | 174.0 |
### Chart
| Category | | | | | | |
|---|---|---|---|---|---|---|MOUSE 3:
Supplementary Fig. 1 (A-C) Normalized T1 signal enhancement curves for individual animals during Gadodiamide injection in healthy tissue and tumour at different time points: before (blue), 24h (red) and 72h (black) after EBRT treatment. (D-F): Quantitative normalized T1 signal enhancement in individual animals.

## Slide 2
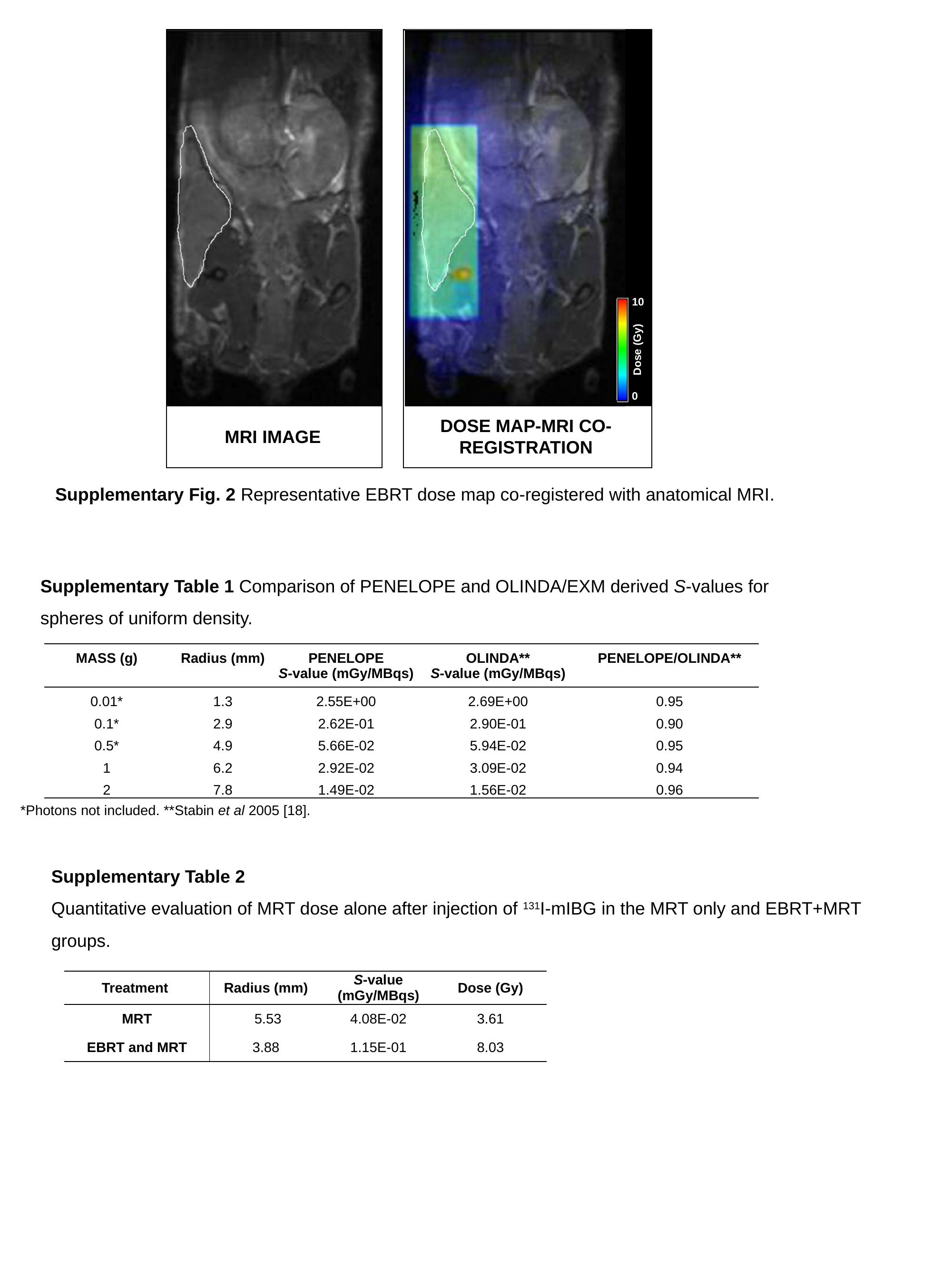

10
Dose (Gy)
0
Dose map-MRI co-registration
MRI image
Supplementary Fig. 2 Representative EBRT dose map co-registered with anatomical MRI.
Supplementary Table 1 Comparison of PENELOPE and OLINDA/EXM derived S-values for spheres of uniform density.
| MASS (g) | Radius (mm) | PENELOPE S-value (mGy/MBqs) | OLINDA\*\* S-value (mGy/MBqs) | PENELOPE/OLINDA\*\* |
| --- | --- | --- | --- | --- |
| 0.01\* | 1.3 | 2.55E+00 | 2.69E+00 | 0.95 |
| 0.1\* | 2.9 | 2.62E-01 | 2.90E-01 | 0.90 |
| 0.5\* | 4.9 | 5.66E-02 | 5.94E-02 | 0.95 |
| 1 | 6.2 | 2.92E-02 | 3.09E-02 | 0.94 |
| 2 | 7.8 | 1.49E-02 | 1.56E-02 | 0.96 |
*Photons not included. **Stabin et al 2005 [18].
Supplementary Table 2
Quantitative evaluation of MRT dose alone after injection of 131I-mIBG in the MRT only and EBRT+MRT groups.
| Treatment | Radius (mm) | S-value (mGy/MBqs) | Dose (Gy) |
| --- | --- | --- | --- |
| MRT | 5.53 | 4.08E-02 | 3.61 |
| EBRT and MRT | 3.88 | 1.15E-01 | 8.03 |
